# Photographic Atlas and Three-Dimensional Reconstruction of the Holotype Skull of *Euhelopus zdanskyi* with Description of Additional Cranial Elements

**DOI:** 10.1371/journal.pone.0079932

**Published:** 2013-11-21

**Authors:** Stephen F. Poropat, Benjamin P. Kear

**Affiliations:** 1 Department of Earth Sciences, Uppsala University, Uppsala, Uppsala County, Sweden; 2 Australian Age of Dinosaurs Natural History Museum, The Jump-Up, Winton, Queensland, Australia; University of Pennsylvania, United States of America

## Abstract

**Background:**

*Euhelopus zdanskyi* is one of relatively few sauropod taxa known from an almost complete skull and mandible. Recent phylogenetic analyses suggest that *Euhelopus* is a somphospondylan titanosauriform, and that it is a member of the clade (Euhelopodidae) which is the sister taxon to the hugely successful, dominantly Cretaceous sauropod group Titanosauria.

**Methodology/Principal Findings:**

The skull elements of *Euhelopus* were CT scanned at Uppsala Akademiska Sjukhuset. Three-dimensional models of the elements were constructed from the DICOM data using Mimics 14.0, InVesalius 3.0, and GeoMagic Studio 2012, the skull was rearticulated in Rhinoceros 4.0, and the final version was rendered in GeoMagic Studio 2012.

**Conclusions/Significance:**

The fact that relatively complete sauropod skulls are so rare in the fossil record, particularly among titanosauriforms, means that the skulls that are known should be as thoroughly described and well-illustrated as possible. This contribution supplements previous descriptions of the cranial elements of *Euhelopus*, one of the few euhelopodid taxa for which cranial material is known, by presenting a comprehensive photographic atlas of the skull elements to facilitate a better understanding of their morphology. We describe several elements which have been overlooked in past studies of *Euhelopus*, and also provide as accurate a reconstruction of the skull as possible (in the absence of the braincase), the most significant components of which are the articulations of the palate and the mandible.

## Introduction

Sauropod skull material is relatively rare in the fossil record. Of the 276 named taxa, 72 (∼26%) are represented by at least one non-dental skull element (Table S1); however, in many cases where cranial remains are present, the skull is very incompletely known (table 1 in [1]). The inclusion of dental material increases the number of species represented by cranial material to 105 of 276 (∼38%), though in several cases these teeth are the only material on which the name is based (electronic supplementary material 5 in [2]). The complete osteology of the skull of the majority of sauropods is not known, and in some cases where a skull is present it has not been described in detail. Taxa which are known from essentially completely known, and more-or-less completely described, skulls and mandibles include: *Shunosaurus lii* Dong et al., 1983 [3]–[7], *Mamenchisaurus youngi* Pi et al., 1996 [8], [9], *Omeisaurus tianfuensis* He et al., 1984 [10], [11] and *Omeisaurus maoianus* Tang et al., 2001 [12] from the Middle Jurassic of China; several species of both *Diplodocus* Marsh, 1878 [1], [13]–[16] and *Camarasaurus* Cope, 1877 [6], [17]–[23] from the Late Jurassic of the United States; *Giraffatitan* (*Brachiosaurus*) *brancai* (Janensch, 1914) Taylor, 2009 [24]–[26] from the Late Jurassic of Tanzania; *Abydosaurus mcintoshi* Chure et al., 2010 [2] from the Early Cretaceous of the United States; *Tapuiasaurus macedoi* Zaher et al., 2011 [27] from the Early Cretaceous of Brazil; and *Nemegtosaurus mongoliensis* Nowiński, 1971 [28]–[30] from the Late Cretaceous of Mongolia. *Melanorosaurus readi* Haughton, 1924 [31] from the Late Triassic of South Africa, the sister taxon to Sauropoda [32], is also known from a complete skull [33]. Other taxa are represented by more-or-less complete cranial remains, including *Amargasaurus cazaui* Salgado and Bonaparte, 1991 [34], [35], *Apatosaurus* Marsh, 1877 [16], [36]–[40], *Bonitasaura salgadoi* Apesteguía, 2004 [41], [42], *Euhelopus zdanskyi* (Wiman, 1929) Romer, 1956 [43]–[46], *Europasaurus holgeri* Sander et al., 2006 [47], *Limaysaurus tessonei* (Calvo and Salgado, 1995) Salgado et al., 2004 [48], [49], *Nigersaurus taqueti* Sereno et al., 1999 [50]–[52], *Patagosaurus fariasi* Bonaparte, 1979 [53]–[55], *Quaesitosaurus orientalis* Kurzanov and Bannikov, 1983 [56], *Rapetosaurus krausei* Curry Rogers and Forster, 2001 [57], [58] and *Tazoudasaurus naimi* Allain et al., 2004 [59]–[61]. However, as McIntosh [62] lamented over two decades ago, many otherwise well-known taxa are not represented by any cranial material at all, notably the basal sauropod *Vulcanodon karibaensis* Raath, 1972 [63], [64], the eusauropod of debatable affinities *Haplocanthosaurus* Hatcher, 1903 [65]–[68], and the derived titanosaur *Opisthocoelicaudia skarzynskii* Borsuk-Białynicka, 1977 [69]. One redeeming feature of the sauropod skull record is that the material is fairly broadly spread both temporally and geographically – at least one Jurassic and two Cretaceous sauropod taxa from each continent (excluding Australasia and Antarctica) are known from cranial elements other than teeth (Table S1).

The skull of *Euhelopus zdanskyi* has received more attention than many sauropod skulls since its initial description [43]–[45]. Nevertheless, some elements have never been figured or described, others have received relatively little attention or only brief descriptions, and most elements have only been illustrated in one or two views. This publication aims to supplement previous studies of *Euhelopus* [43]–[45] by re-illustrating all of the preserved elements and presenting a three-dimensional reconstruction of the skull from computed tomographic scans, thereby fully illustrating the articulations of the skull elements and providing the reader with the most accurate depiction of the skull of *Euhelopus* to date.

The importance of this work is augmented by the controversy which, until recently, surrounded the phylogenetic placement of *Euhelopus* within Sauropoda. Some works supported a close relationship with *Mamenchisaurus*, *Omeisaurus*, and *Shunosaurus* [70]–[73], forming the clade Euhelopodidae, whereas others resolved it as the sister taxon to Titanosauria [74], [75]. A full redescription of *Euhelopus*, and a thorough revision of the character coding for two matrices which had previously produced conflicting results, led to both resolving *Euhelopus* as the sister taxon to Titanosauria, within the clade Somphospondyli [45]. The resurrection of a redefined Euhelopodidae (excluding the Jurassic *Mamenchisaurus*, *Omeisaurus* and *Shunosaurus*, and restricted to Asian Cretaceous taxa) has been proposed recently [76], though the taxa comprising this clade as defined therein were not all resolved in Euhelopodidae in a subsequent study of titanosauriform inter-relationships [77. The currently accepted phylogenetic placement of *Euhelopus* as being closely related to Titanosauri, the only sauropod group to have persisted until the end of the Cretaceous, underlines the importance of achieving as full an understanding of the anatomy of *Euhelopus* as possible.

### Institutional Abbreviations

Dinosaur National Monument, Utah, USA: DNM; Palaeontological Museum, Museum of Evolution [Evolutionsmuseet Paleontologi], Uppsala, Sweden: PMU.

### Historical Background

**Figure 1:**
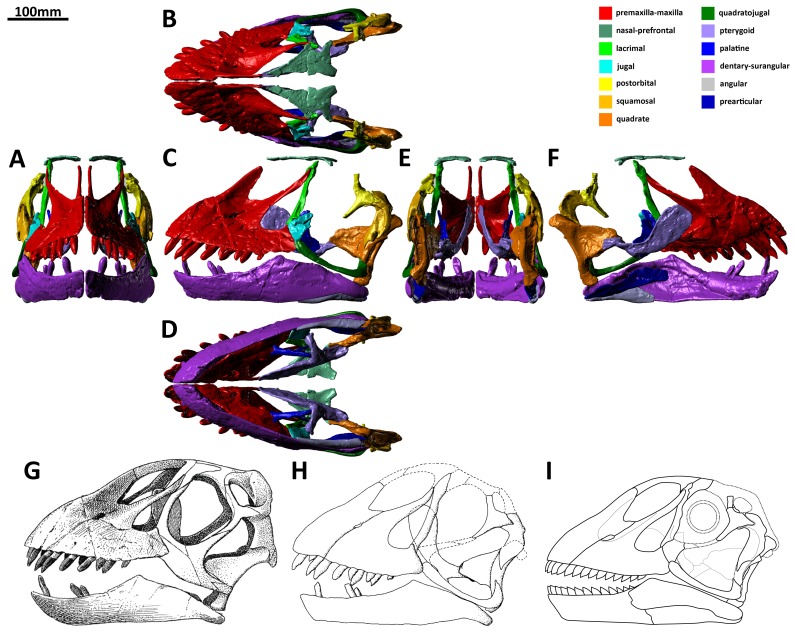
Reconstruction of the skull of *Euhelopus zdanskyi* based on the holotype elements (PMU 24705/1 [formerly PMU R 233]) from “exemplar a” in rostral (A), dorsal (B), left lateral (C), ventral (D), caudal (E), and medial (F) views; the right side of the skull has been removed in medial view to show the organisation of the palatal and mandibular elements. More complete and/or better preserved elements were used in this reconstruction when both left and right elements were preserved. The reconstructions of the skull provided by Wiman [43] (G) and Mateer and McIntosh [44] (H) are presented, as is a line drawing of the skull as reconstructed in this work (I).

*Euhelopus zdanskyi* was the first sauropod (and, indeed, one of the first non-avian dinosaurs [45]) described from China and is one of relatively few titanosauriforms to preserve a relatively complete skull (Figure 1). The skull was first described by Wiman [43], who misinterpreted some elements and did not mention others. The majority of the remaining cranial remains were described by Mateer and McIntosh [44], and Wilson and Upchurch [45] made further observations on several elements. A bone-by-bone breakdown of the descriptions and figures of the holotype cranial (and postcranial) elements given in these three papers is presented in Table S2.

Despite the fact that so much work on the skull of *Euhelopus* has already been conducted, research in the PMU collections has revealed that there are several skull elements and fragments that have not been mentioned in any previous studies; indeed, some have not even been fully prepared from the matrix in which they were preserved. The following bones have been identified in the collections: the rostral portion of the left nasal (PMU 24705/1c [formerly PMU R 233 v]); the partial right jugal (PMU 24705/1u [formerly PMU R 233 u]); the tapered jugal process of the postorbital (PMU 24705/1v [formerly PMU R 233 y]), still partially encased in matrix; the dorsal process of the right quadratojugal (PMU 24705/1s [formerly PMU R 233 x]); and the fragmented left pterygoid (comprised of PMU 24705/1l [formerly PMU R 233 ä] and PMU 24705/1w [formerly PMU R 233 ö]). An additional fragment, PMU 24705/1t [formerly PMU R 233 p], has proven more difficult to place; it may represent the right splenial, though it is not possible to be certain of this identification as it is too fragile to be prepared from the encasing matrix.

The holotype specimen of *Euhelopus zdanskyi*, which includes all of the known skull material, was formerly catalogued as PMU R 233 but is now catalogued as PMU 24705 [78]. Aside from the skull elements which were put on display (which were catalogued together as PMU R 233 a), each individual specimen was assigned a letter following the number. Both numbering sequences are employed throughout this paper, and a full list of the specimens which comprise the holotype of *Euhelopus zdanskyi* (with both old and new specimen numbers) can be found in Table S2.

## Methods

J. O. R. Ebbestad of the Evolutionsmuseet, Uppsala granted us access to the skull elements of *Euhelopus*. These were CT scanned by M. Segelsjö at Uppsala Akademiska Sjukhuset. It was hoped that there would be a sufficient density difference between the bones and the plaster and matrix to facilitate their digital separation, but this was not the case. As such, all elements used in the final skull reconstruction (Figure 1; Figure S1) have been left as they are preserved and/or reconstructed, with the exception of the pterygoid, which was digitally separated from the quadrate.

DICOM (Digital Imaging and Communications in Medicine) data were obtained from the CT scanner and processed in Mimics v14.0 and InVesalius 3.0. Surface models of all elements were generated in these programs, though several meshes were repaired in GeoMagic Studio 2012. The elements were rearticulated in Rhinoceros 4.0, and the final 3-D model presented herein as a supplementary figure was exported as a three-dimensional PDF (Figure S1) from GeoMagic Studio 2012.

## Results and Discussion

### Specimen Descriptions

#### Premaxilla and maxilla (PMU 24705/1a [formerly PMU R 233 a]: Figure 2; and PMU 24705/1b [formerly PMU R 233 a]: Figure 3)

**Figure 2:**
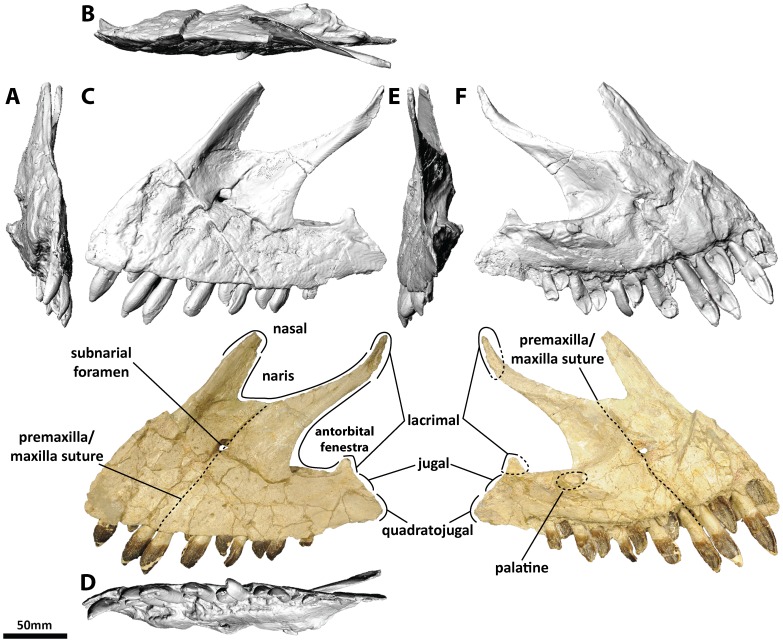
Left premaxilla and maxilla (PMU 24705/1a [formerly PMU R 233 a]) of *Euhelopus zdanskyi* in rostral (A), dorsal (B), left lateral (C), ventral (D), caudal (E), and medial (F) views. Note the distinctive buttresses on the lingual side of the teeth, located near the base of the crown.

**Figure 3:**
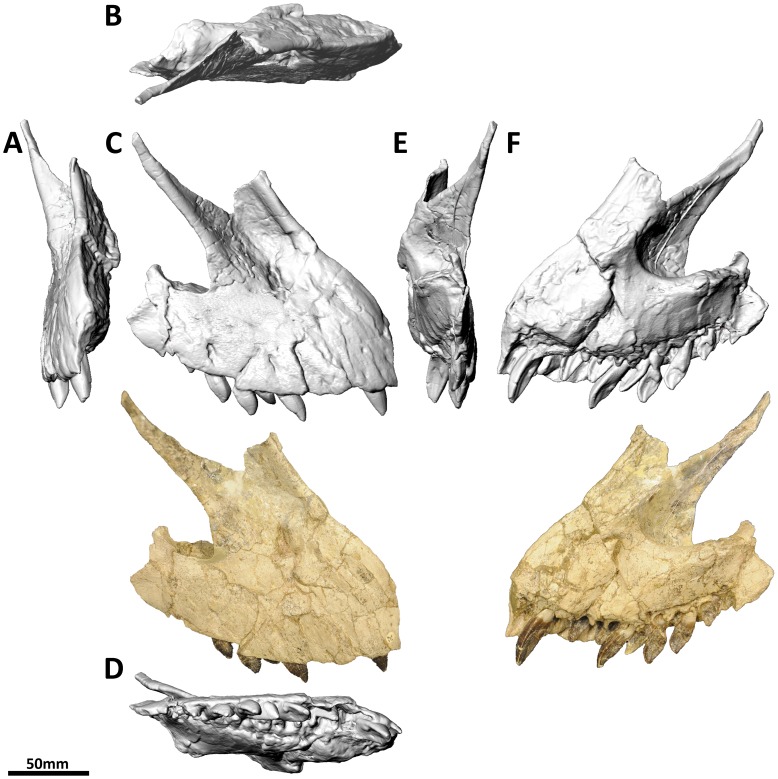
Right premaxilla and maxilla (PMU 24705/1b [formerly PMU R 233 a]) of *Euhelopus zdanskyi* in rostral (A), dorsal (B), right lateral (C), ventral (D), caudal (E) and medial (F) views.

Few additions to the description of the premaxillae and maxillae provided by Wilson and Upchurch [45] are necessary. Based on the CT data, the premaxilla-maxilla contact is planar in lateral view. It should be noted that Wilson & Upchurch (figure 6 in [45]) actually figured the left premaxilla-maxilla reversed in medial view, not the right premaxilla-maxilla as stated in the caption.

The left premaxilla-maxilla is 23.75 cm long (measured along the tooth margin) as preserved, while the right is 16.67 cm long. The medial views of these elements (Figures 2F and 3F) highlight this discrepancy clearly, with the medial expansion of the antorbital fenestra (behind the ascending process of the maxilla) expressed as a much deeper concavity on the right maxilla than on the left. Precisely which of these elements most closely approximates the original morphology of the premaxilla-maxilla is unclear; however, the fact that the teeth have been pushed out of their sockets in the left premaxilla-maxilla (based on the extent of the enamel on the teeth) suggests that it is the right which has undergone less deformation. However, it is more difficult to accommodate the right premaxilla-maxilla into a reconstruction of the skull than the left, and it is clear that it has also been deformed. As such, the left premaxilla-maxilla, which has a better preserved premaxillary ascending process and caudal jugal process, has been used as the representative element in our three-dimensional skull reconstruction (Figure 1A–F; Figure S1); the same element was used in the line drawing of the skull (Figure 1G), though the anterior portion was compressed to compensate for distortion.

#### Nasal and Prefrontal (PMU 24705/1c [formerly PMU R 233 v] and PMU 24705/1d [formerly PMU R 233 t]: Figure 4)

**Figure 4:**
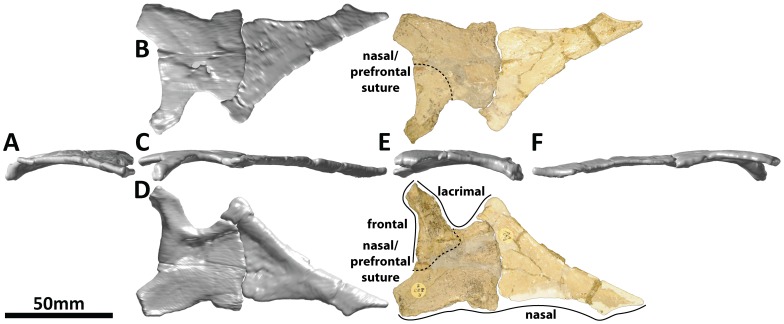
Right nasal and partial right prefrontal (PMU 24705/1c [formerly PMU R 233 v] and PMU 24705/1d [formerly PMU R 233 t]) of *Euhelopus zdanskyi* in rostral (A), dorsal (B), right lateral (C), ventral (D), caudal (E), and medial (F) views. White portions on the rostral process and grey patches on the caudal portion have been reconstructed with plaster.

PMU 24705/1d [formerly PMU R 233 t] was interpreted as the right frontal by Mateer and McIntosh [44] and as the left nasal by Wilson and Upchurch [45]. We agree that this element is a nasal; however, we have discovered that an element in the collection (PMU 24705/1c [formerly PMU R 233 v]) represents the rostral portion of this bone. This identification means that this element is in fact the right nasal, to which at least part of the right prefrontal remains solidly sutured at the caudomedial margin. This reinterpretation necessitates a full redescription of this element.

The nasal is, in many respects, very similar to the nasal of *Camarasaurus* [21], [22]. However, the broken state of the bone, the orientation of the lateral process, and the fact that the bone has been flattened (a fact which becomes apparent when this element is compared with the nasal of *Camarasaurus lentus* DNM 28 (fig. 12A in [21])), demonstrate that it has suffered post mortem deformation. The lateral process, which would have articulated with the dorsal portion of the lacrimal in life, is only incompletely preserved, has been restored with plaster, and should be oriented ventrolaterally rather than laterally. Portions of the rostral process of the nasal have been reconstructed or otherwise altered with plaster, meaning that when this element is digitally reflected, a large gap is present between the paired nasals along the midline. A small gap between the premaxillary processes of the nasals of *Camarasaurus* has been noted previously [21], though in *Euhelopus* the gap has probably been exaggerated by post mortem deformation: the flattening of this element has altered the course of the medial margin of the rostral process.

The nasal is slightly concave on its ventral surface and correspondingly convex on its smooth dorsal surface. The lateral process is supported by a subtle ventral ridge which bifurcates at the base, with one branch following the narial margin and the other directed towards the prefrontal as in *Camarasaurus* [21]. The nasal would not have contributed to the orbit, being excluded by the prefrontal, as in other sauropods (fig. 16.2 in [62]).

At least part of the right prefrontal appears to be sutured to the caudal margin of the nasal. This was determined from comparisons with that of *Camarasaurus lentus* (DNM 28 (fig. 12C in [21])): the morphology of the ridges on the ventral surfaces of both nasals correspond well only if the caudal-most portion of PMU 24705/1d is not part of the nasal. A more detailed description of the prefrontal cannot be given due to both its incomplete nature and post mortem deformation.

#### Lacrimal (PMU 24705/1e [formerly PMU R 233 α]: Figure 5; and PMU 24705/1f [formerly PMU R 233 β]: Figure 6)

**Figure 5:**
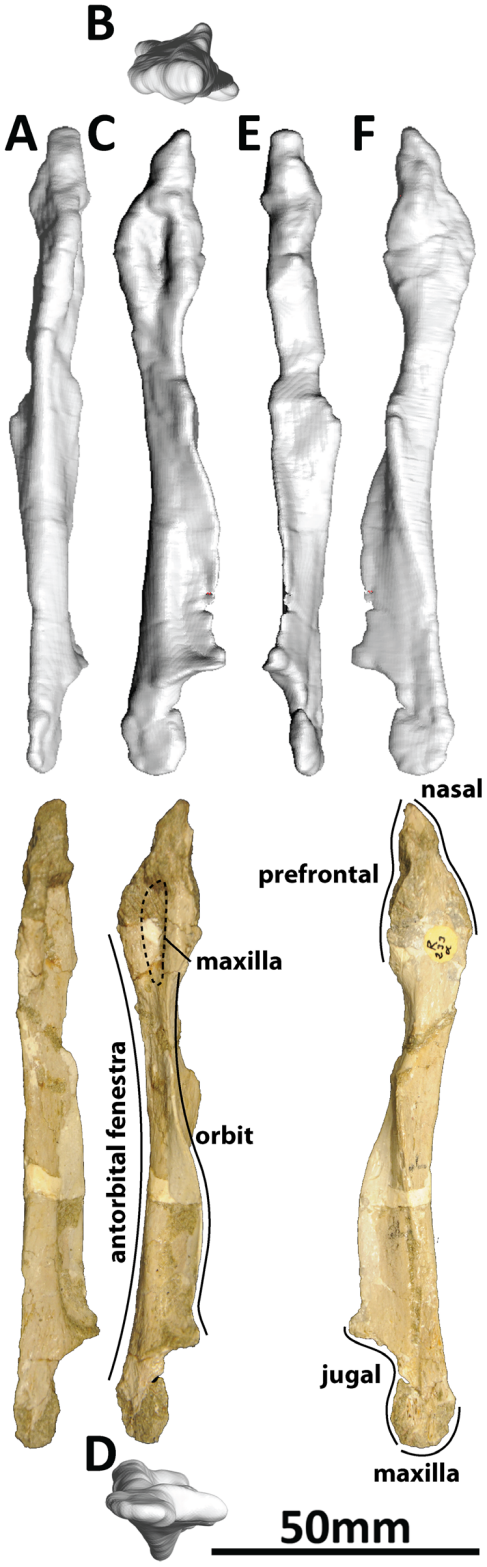
Left lacrimal (PMU 24705/1e [formerly PMU R 233 α]) of *Euhelopus zdanskyi* in rostral (A), dorsal (B), left lateral (C), ventral (D), caudal (E), and medial (F) views.

**Figure 6:**
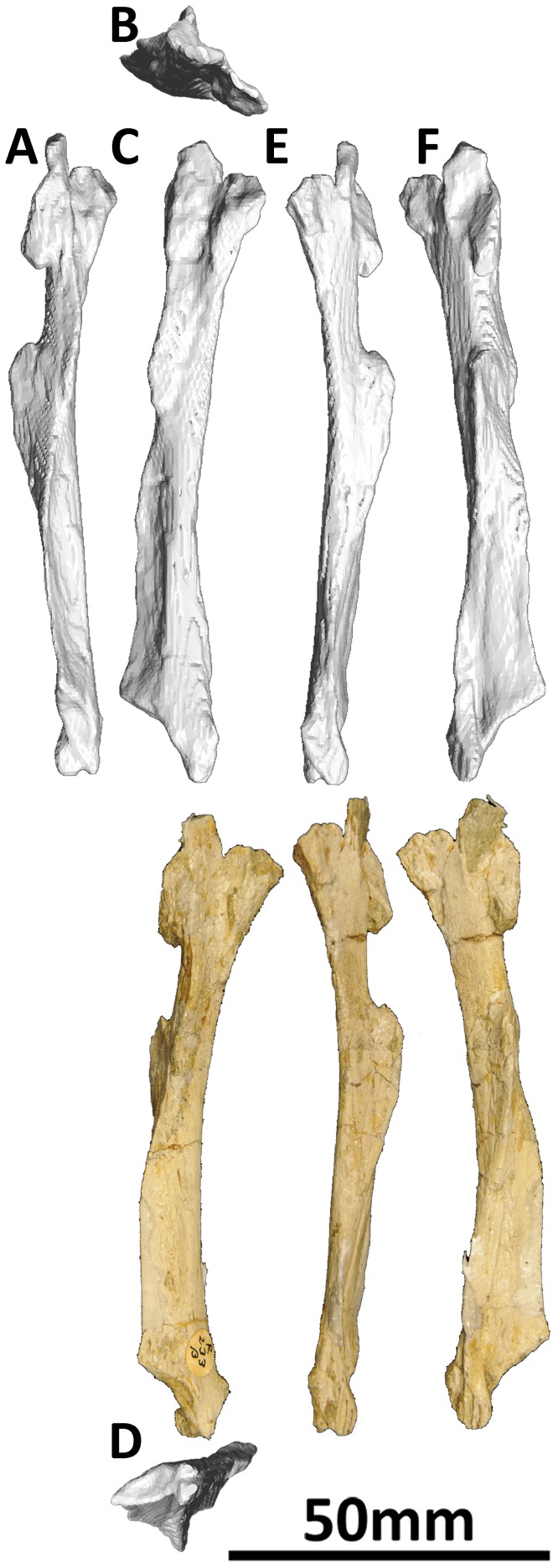
Right lacrimal (PMU 24705/1f [formerly PMU R 233 β]) of *Euhelopus zdanskyi* in rostral (A), dorsal (B), right lateral (C), ventral (D), caudal (E), and medial (F) views.

The lacrimal was one of the few elements not commented upon by Wilson and Upchurch [45]. Mateer and McIntosh [44], in their description of the lacrimal, state that the right lacrimal is complete; however, it is the left that is more complete, as their figure label correctly states (fig. 3 a–d in [44]). The left lacrimal is illustrated upside-down in Mateer and McIntosh’s figures (fig. 3 c–d in [44]).

The lacrimal is a slender bone, oriented approximately vertically. Dorsally, it articulates with the ascending process of the maxilla, the lateral surface of the nasal, and the rostral surface of the prefrontal, whereas ventrally it abuts the caudal processes of the maxilla and the rostral surface of the jugal. The antorbital fenestra was completely enclosed by the maxilla and lacrimal, as suggested by previous authors [44], [45].

The dorsal portion of the lacrimal is diamond shaped in lateral view, is the transversely thickest portion of the element, and bears a lateral fossa to accommodate the medial surface of the maxillary ascending process. The nasal would have articulated on the rostral face of the dorsal portion of the lacrimal, while the prefrontal would have abutted the caudal margin. The shaft of the lacrimal is thickest on its rostral margin, which would have formed the caudal rim of the antorbital fenestra. A thin but extensive ridge projects caudolaterally from the shaft on the ventral half of the lacrimal, whereas a small bulge projects caudomedially at approximately one-third of the length from the dorsal margin. The ventral end of the lacrimal is transversely compressed, and bears a caudoventrally facing concave surface to accommodate the rostrodorsal portion of the jugal. The ventral-most portion of the lacrimal articulates with a ridge on the medial surface of the maxilla, which would also have accommodated the rostral-most portion of the jugal.

The lacrimal foramen was not able to be observed on either specimen, even in the CT data – this may be due to deformation of both elements.

#### Jugal (PMU 24705/1u [formerly PMU R 233 u]: Figure 7)

**Figure 7:**
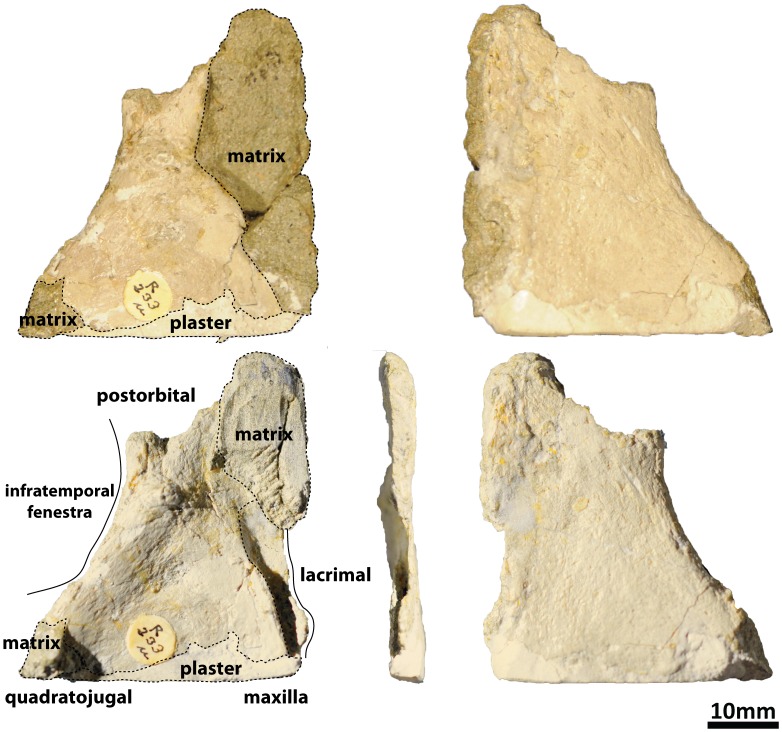
Right jugal (PMU 24705/1u [formerly PMU R 233 u]) of *Euhelopus zdanskyi* in lateral (A) and medial (B) views prior to additional preparation, and in lateral (C), anterior (D) and medial (E) views after additional preparation. As is clearly shown by comparing the two sets of photographs, the most distinctive feature of this element, the groove for articulation with the lacrimal, was only revealed after additional preparation.

PMU 24705/1u was never mentioned in previous studies of the skull of *Euhelopus* and, until recently, had never been fully prepared. The element is broadly triangular, with two of the margins essentially complete and the other incomplete and restored with plaster. One side represents finished bone, with no notable features; the other revealed, following the removal of sediment, the presence of a narrow, shallow groove. The presence of this groove, and the overall similarity of this element to the jugal of *Giraffatitan brancai* ( [25] abb. 16), strongly suggests that PMU 24705/1u represents an incomplete right jugal.

The plastered margin is interpreted as the incomplete ventral margin, which would have articulated with the dorsal margin of the caudal process of the maxilla rostrally and the dorsal margin of the rostral process of the quadratojugal caudally; consequently, the jugal would have been excluded from the ventral margin of the cranium (as interpreted by Mateer and McIntosh [44]). The ventral portion of the jugal would probably have extended further caudally as seen in *Giraffatitan* [25]. The grooved margin would have articulated with the lacrimal, as in *Giraffatitan* [25], with the medial lip of the groove extending further rostrally than the lateral. The smooth, finished margin (which is also the transversely thickest of those preserved) is interpreted as the caudal margin, and this would have formed the rostral rim of the infratemporal fenestra. The dorsal part of the jugal, between the rostral and caudal margins, would have had a caudodorsally/rostroventrally angled process meeting the jugal process of the postorbital.

It is quite likely that, when complete, the jugal of *Euhelopus* bore a closer resemblance to that of *Giraffatitan* than to any other sauropod based on the similar grooved morphology of the rostral margin in both.

As can be seen in the three-dimensional model of the skull (Figure S1), the jugal is laterally concave (and correspondingly medially convex) and does not seem to articulate well with the maxilla. Thus, we suggest that this element was distorted post mortem.

#### Postorbital (PMU 24705/1 g [formerly PMU R 233 a], PMU 24705/1v [formerly PMU R 233 y]: Figure 8)

**Figure 8:**
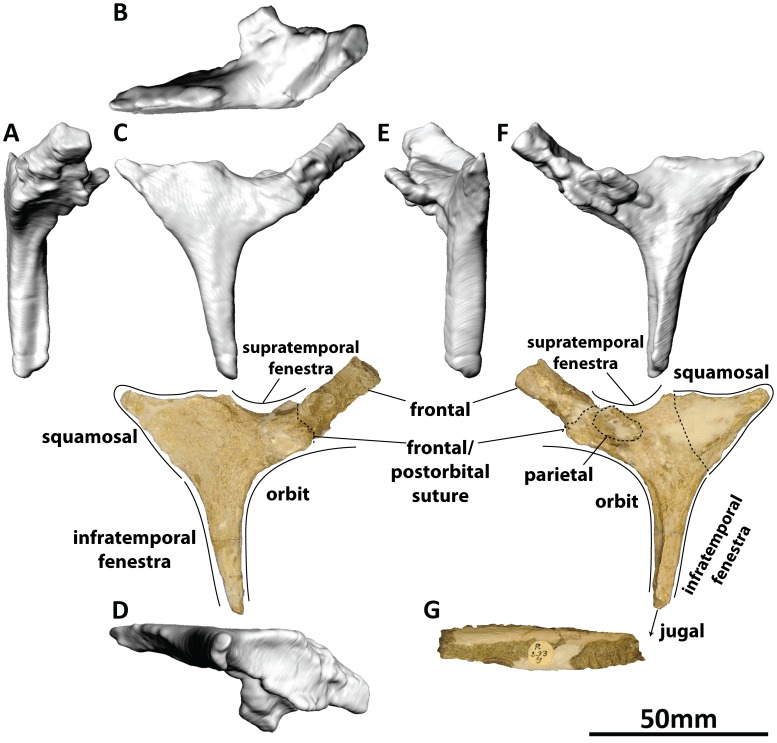
Right postorbital (PMU 24705/1 g [formerly PMU R 233 a]) of *Euhelopus zdanskyi* in rostral (A), dorsal (B), right lateral (C), ventral (D), caudal (E), and medial (F) views; and PMU 24705/1v (formerly PMU R 233 y), a portion of bone still partially embedded in matrix which may be the tapered end of the jugal process of the postorbital (G).

The right postorbital is present in the holotype specimen of *Euhelopus*, not the left as stated by Wilson and Upchurch in their specimen list [45]. However, the description provided by these authors (which does not specify the side from which the postorbital derived) clearly reflects that the authors described it (correctly) as the right postorbital; thus, it would appear that the error in the specimen list is typographic. The jugal process would have been significantly longer in life; indeed, a thin fragment of bone still partially embedded in matrix (PMU 24705/1v [formerly PMU R 233 y]) may represent the termination of this process. The postorbital is very similar to the corresponding element in *Giraffatitan brancai* [25], and no emendations to the description of this element by Wilson and Upchurch [45] are necessary.

#### Squamosal (PMU 24705/1 h [formerly PMU R 233 a]: Figure 9)

**Figure 9:**
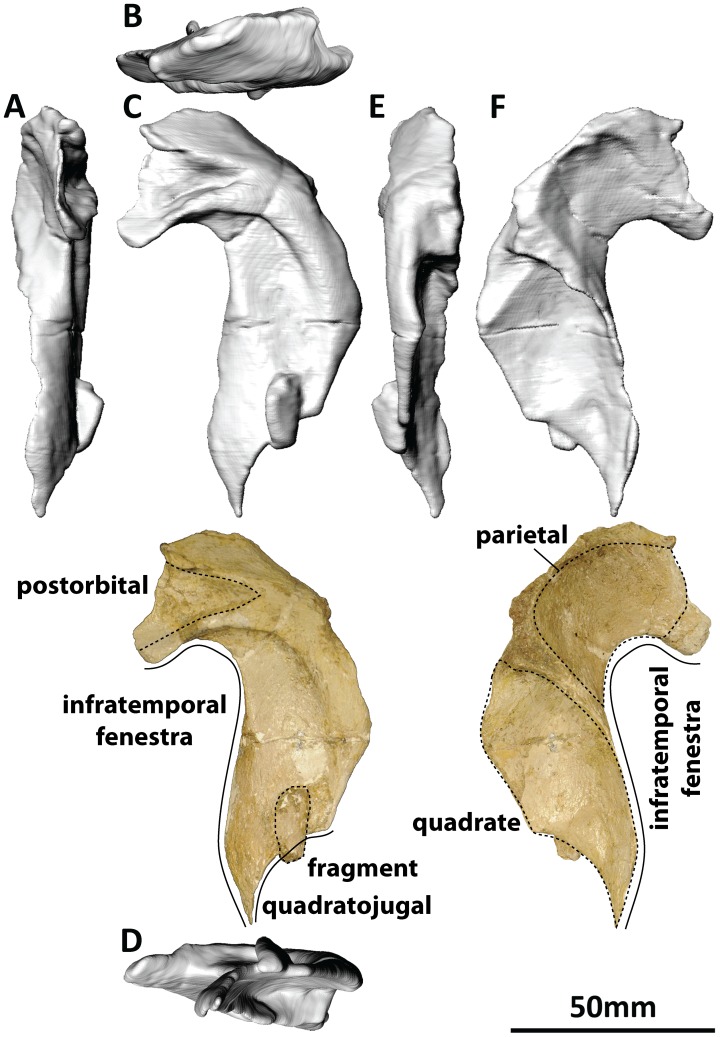
Left squamosal (PMU 24705/1 h [formerly PMU R 233 a]) of *Euhelopus zdanskyi* in rostral (A), dorsal (B), left lateral (C), ventral (D), caudal (E), and medial (F) views.

The left squamosal is incompletely preserved, as stated by Wiman [43] and Wilson and Upchurch [45] (*contra* Mateer and McIntosh [44]). Digital mirror-imaging of the squamosal (which was necessary because the only preserved squamosal is the left and the only preserved quadrate is the right) allows perfect articulation with the dorsolateral region of the quadrate. The description of the squamosal provided by Wilson and Upchurch [45] is thorough and accurate, and it is similar in shape to those of *Giraffatitan brancai* [25], *Mamenchisaurus youngi* [9], and *Camarasaurus* sp. [21]. The bulge that can be seen on the lateral surface of the squamosal is a broken fragment of bone.

#### Quadratojugal (PMU 24705/1i [formerly PMU R 233 z]: Figure 10; and PMU 24705/1j [formerly PMU R 233 å]: Figure 11)

**Figure 10:**
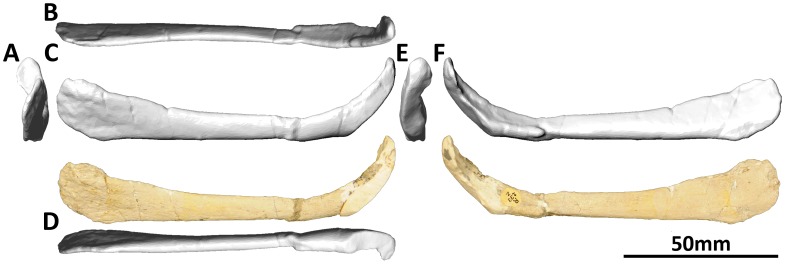
Left quadratojugal (PMU 24705/1i [formerly PMU R 233 z]) of *Euhelopus zdanskyi* in rostral (A), dorsal (B), left lateral (C), ventral (D), caudal (E), and medial (F) views.

**Figure 11:**
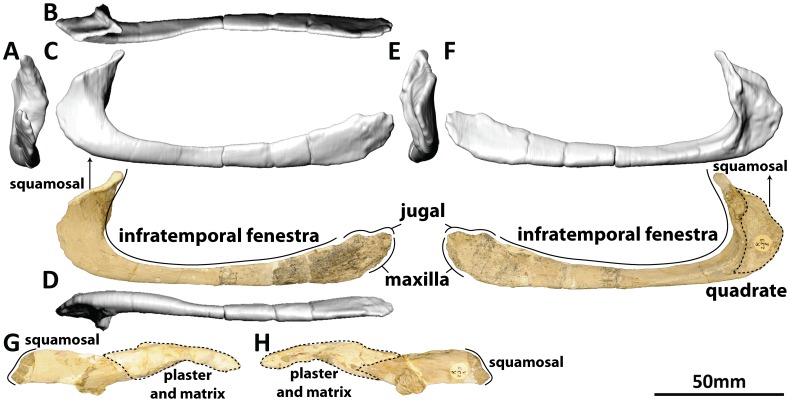
Right quadratojugal (PMU 24705/1j [formerly PMU R 233 å]) of *Euhelopus zdanskyi* in rostral (A), dorsal (B), left lateral (C), ventral (D), caudal (E), and medial (F) views; and the dorsal process of the right quadratojugal (PMU 24705/1j [formerly PMU R 233 å]) in lateral (G) and medial (H) views; in lateral view, the dorsal end is to the left, while in medial view the dorsal end is to the right.

The quadratojugals of *Euhelopus* were first recognised by Mateer and McIntosh [44] and were thoroughly described by Wilson and Upchurch [45]. However, we have determined that an isolated element in the PMU collection, PMU 24705/1s (formerly PMU R 233 x), represents the dorsal process of the right quadratojugal. The dorsal margin of this process would have articulated with the ventral extension of the squamosal, excluding the quadrate from the lateral margin of the infratemporal fenestra, as in *Giraffatitan brancai* [25] and *Camarasaurus* sp. [21]. As in *Giraffatitan* [25], a roughened, ∼15 mm long surface on the dorsal margin of the rostral end of the quadratojugal represents the articular surface of the jugal, whereas the rostral surface of the rostral end would have abutted the caudal surface of the caudal portion of the maxilla.

#### Quadrate (PMU 24705/1k [formerly PMU R 233 a]: Figure 12)

**Figure 12:**
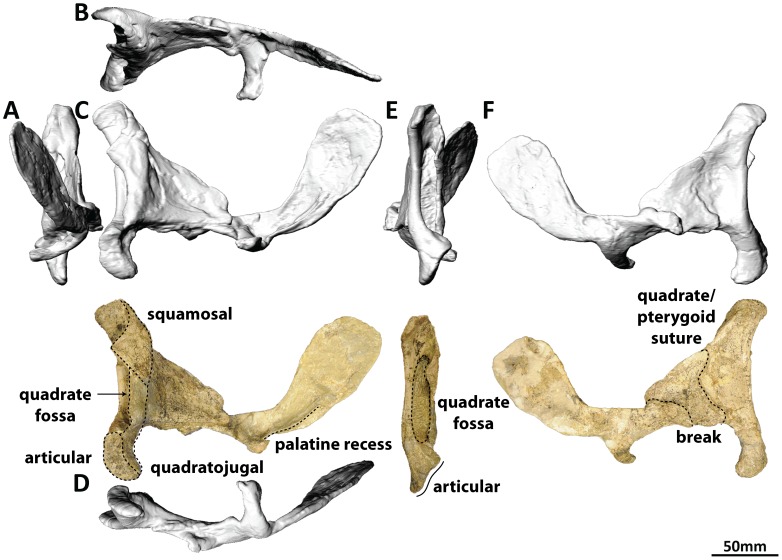
Right quadrate, pterygoid, and vomer (PMU 24705/1k [formerly PMU R 233 a]) of *Euhelopus zdanskyi* in rostral (A), dorsal (B), right lateral (C), ventral (D), caudal (E), and medial (F) views.

The quadrate was fully described by Wilson and Upchurch [45]. As stated above, the squamosal can be shown to perfectly articulate with the medial expansion of the lateral wall of the caudal fossa of the quadrate; thus, Mateer and McIntosh [44] positioned the squamosal slightly too high relative to the quadrate in their skull reconstruction. As preserved, the ventral articular surface faces ventrolaterally. Wilson and Upchurch [45] suggested that this surface was incomplete; however, based on the texture of the bone, and the fact that similarly bevelled ventral articular surfaces have also been observed in quadrates of *Camarasaurus* (fig. 20 H, L, T, X in [21]), we interpret this surface as complete. This would mean that the articular surface of the articular, had it been preserved, would have faced dorsomedially.

#### Right pterygoid (PMU 24705/1k [formerly PMU R 233 a]: Figure 12)

The precise morphology of the pterygoid is hard to discern and the angle at which it was reconstructed by Wiman [43] is far too steep, as noted by Mateer and McIntosh [44]. When the rostral portion of the pterygoid was detached (digitally) from the quadrate and rotated so that it was more horizontal than preserved, it was found that the rostral tip came close to contacting the internal surface of the premaxilla-maxilla junction, at the intersection of the bases of the ascending processes of the maxillae. In all sauropods, including *Diplodocus* [16], [75], *Giraffatitan* [25] and *Camarasaurus* [21], the rostral processes of the pterygoids are wedged between the vomers [62], [73], which in turn abut the premaxillae/maxillae. Direct observation of the right pterygoid suggests that part of the right vomer might be fused to the rostral process of the right pterygoid, thereby explaining the otherwise strange morphology of this element. However, the methods employed in the restoration of this element in the 1920s mean that identifying the boundary between the potentially present vomer and the pterygoid is not possible, even using the CT data.

#### Left pterygoid (PMU 24705/1l [formerly PMU R 233 ä], PMU 24705/1w [formerly PMU R 233 ö]: Figure 13)

**Figure 13:**
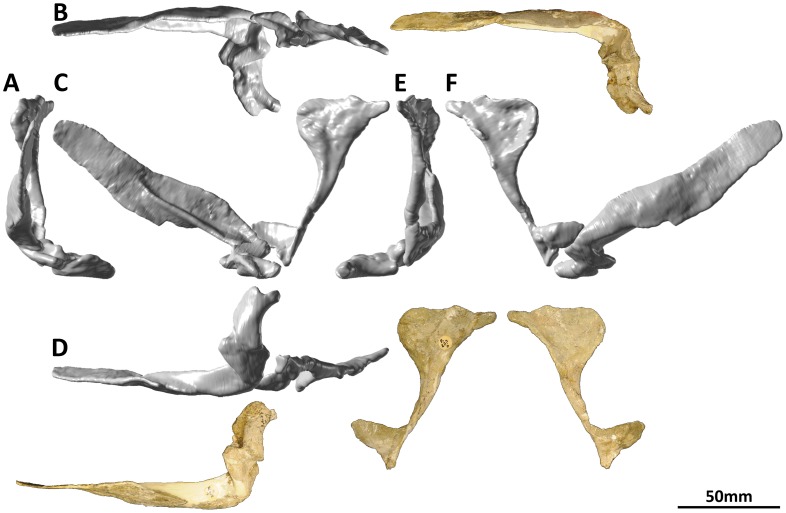
Reconstructed left pterygoid (PMU 24705/1l [formerly PMU R 233 ä] and PMU 24705/1w [formerly PMU R 233 ö]) of *Euhelopus zdanskyi* in rostral (A), dorsal (B), left lateral (C), ventral, (D), caudal (E), and medial (F) views. Note that the point of contact between the two portions is offset – the two elements no longer attach perfectly due to post mortem deformation of the element.

The left pterygoid of *Euhelopus*, though not mentioned by previous authors, is present in the PMU collection. PMU 24705/1l (formerly PMU R 233 ä) has been heavily reconstructed but clearly shows a similar morphology to the right pterygoid/vomer (PMU 24705/1k [formerly PMU R 233 a]) in having a broad, elongate rostral plate and a laterally projecting transverse process.

Another fragmentary skull element in the PMU collection also attaches to PMU 24705/1l: PMU 24705/1w (formerly PMU R 233 ö) can be shown to attach to the caudodorsal surface of PMU 24705/1l; however, as a result of either post mortem deformation or restoration errors, the articulation between the two is imperfect. If correctly identified, the broad, semi-circular plate of PMU 24705/1w is the quadrate articulation facet of the left pterygoid. In comparison with the right pterygoid, the connection between the quadrate articulation and the lateral and rostral projections of the left pterygoid is very thin, possibly a result of distortion of the element.

As preserved and reconstructed, the left pterygoid is not a perfect match for the right pterygoid. The rostrocaudal lengths of both are comparable, whereas the dorsoventral heights vary greatly, the right being approximately twice as tall. The palatine ridge, situated on the caudal portion of the rostral process, has been reconstructed and heavily exaggerated in the left pterygoid; thus, the morphology of this feature is more accurately conveyed in the right pterygoid. Since no trace of the vomer can be observed on the left pterygoid, this element more accurately reflects the morphology of the rostral portion of the pterygoid of *Euhelopus*.

#### Palatine (PMU 24705/1 m [formerly PMU R 233 δ]: Figure 14; and PMU 24705/1n [formerly PMU R 233 γ]: Figure 15)

**Figure 14:**
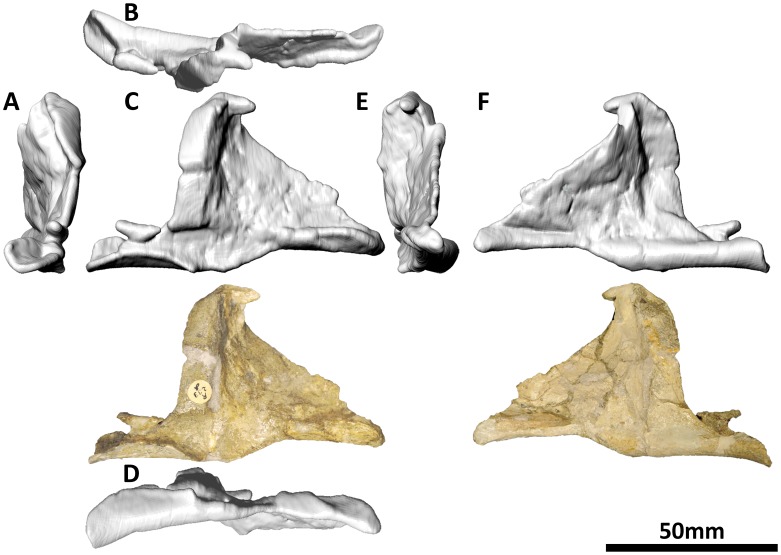
Left palatine (PMU 24705/1 m [formerly PMU R 233 δ]) of *Euhelopus zdanskyi* in rostral (A), dorsal, (B), left lateral (C), ventral (D), caudal (E), and medial (F) views.

**Figure 15:**
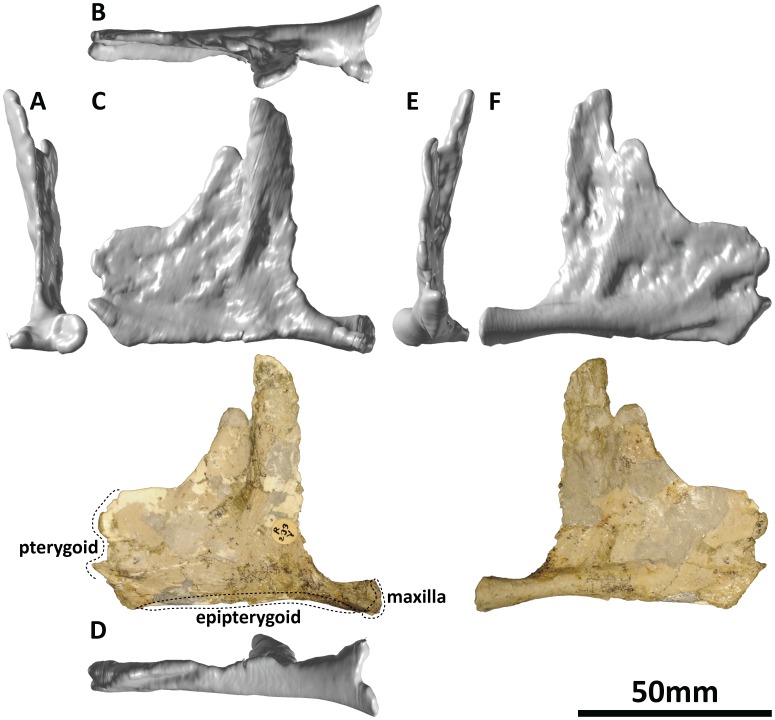
Right palatine (PMU 24705/1n [formerly PMU R 233 γ]) of *Euhelopus zdanskyi* in rostral (A), dorsal (B), right lateral (C), ventral (D), caudal (E), and medial (F) views.

The palatines of *Euhelopus* were originally misidentified as vomers [43] but were later correctly identified [25]. They have been thoroughly described [44], [45], though one additional observation can be made regarding their articulation within the palate: a small bulge on the medial surface of the left maxilla, immediately ventral to the rostroventral margin of the external antorbital fenestra, appears to represent a tiny portion of the rostral process of the left palatine. This location corresponds well with the articulation facet for the palatine on the maxilla of *Giraffatitan* [25], though in this taxon no bulge is present, supporting the notion that this feature in *Euhelopus* is actually a small part of the palatine.

#### Dentary (PMU 24705/1o [formerly PMU R 233 a]: Figure 16; and PMU 24705/1p [formerly PMU R 233 a]: Figure 17)

**Figure 16:**
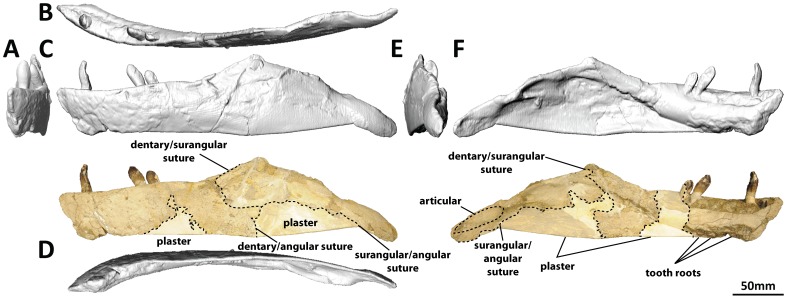
Left dentary and surangular (PMU 24705/1o [formerly PMU R 233 a]) of *Euhelopus zdanskyi* in rostral (A), dorsal (B), left lateral (C), ventral (D), caudal (E), and medial (F) views. Note that the area which would have accommodated the angular has been reconstructed with plaster and painted; other white patches also represent areas that have been reconstructed in plaster.

The description of the dentaries provided by Wilson and Upchurch [45] was comprehensive. Digital scans have confirmed that each dentary has 13 alveoli. The preserved teeth in the left dentary are the second, seventh and eighth; the first tooth, though only slightly erupted, can be observed rostral to the second tooth in medial view (Figure 16F). The roots of several of the rostral-most teeth can be observed on the eroded ventral surface of the dentary as well (Figure 16F).

#### Surangular (PMU 24705/1o [formerly PMU R 233 a]: Figure 16; and PMU 24705/1p [formerly PMU R 233 a]: Figure 17)

**Figure 17:**
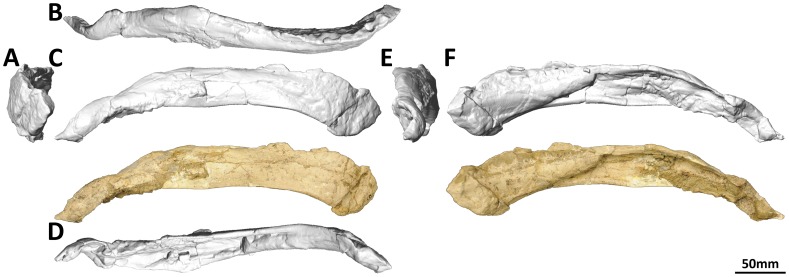
Right dentary and surangular (PMU 24705/1p [formerly PMU R 233 a]) of *Euhelopus zdanskyi* in rostral (A), dorsal (B), lateral (C), ventral (D), caudal (E), and medial (F) views.

Both surangulars are poorly preserved and fused to their respective dentaries. It is probable that the surangular was taller than the angular, though deformation of both mandibles renders this interpretation difficult to make.

#### Angular (PMU 24705/1q [formerly PMU R 233 s]: Figure 18)

**Figure 18:**
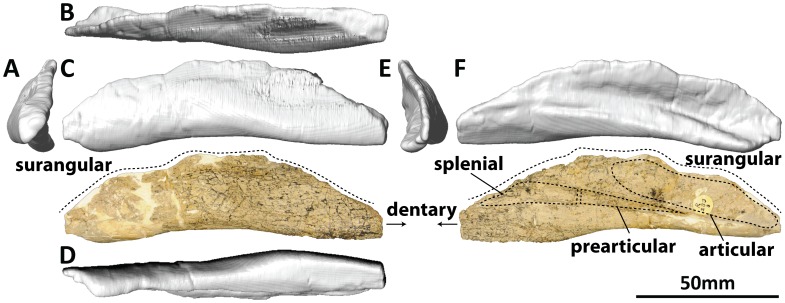
Right angular (PMU 24705/1q [formerly PMU R 233 s]) of *Euhelopus zdanskyi* in rostral (A), dorsal (B), right lateral (C), ventral (D), caudal (E), and medial (F) views.

The angular has been thoroughly described [45] and will not be commented on further herein, though it should be noted that we consider the caudal portion to be essentially complete, whereas the rostral portion is largely missing (and possibly still attached to the right dentary-surangular (Figure 17D, F). The loose angular in the collection (PMU R 233 s) is the right as interpreted by Mateer and McIntosh [44], not the left, as described by Wilson and Upchurch [45]. This is most clearly demonstrated at the caudal margin, where the dorsomedially facing concavity of the angular broadens considerably to accommodate the (missing) articular.

#### Prearticular (PMU 24705/1r [formerly PMU R 233 r]: Figure 19)

**Figure 19:**
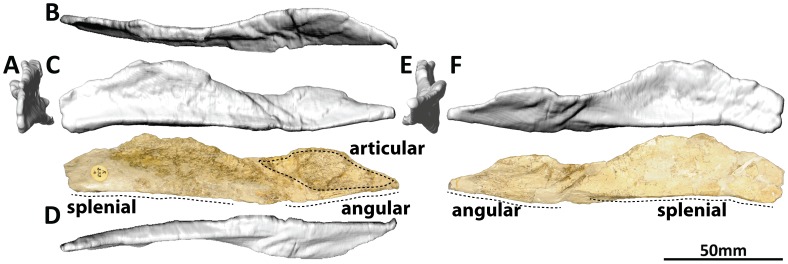
Left prearticular (PMU 24705/1r [formerly PMU R 233 r]) of *Euhelopus zdanskyi* in rostral (A), dorsal (B), left lateral (C), ventral (D), caudal (E), and medial (F) views.

The only prearticular described to date for *Euhelopus zdanskyi* was identified as the left [44], [45], and this identification is agreed with herein; however, it should be noted that Mateer and McIntosh’s figure caption (fig. 4 A–B in [44]) incorrectly identifies this element as the right prearticular.

The prearticular was not described in detail by Mateer and McIntosh [44], who stated that it was similar to the corresponding element in *Camarasaurus*, and no comments were made by Wilson and Upchurch [45]. The authors of the current work would have had great difficulty determining the manner of articulation of this element with the surangular and the angular without Janensch’s reconstructions of the mandible of *Giraffatitan brancai* ( [25] p. 174, Abb. 44–49). The cross-sections and dorsal views of the mandible provided by Janensch [25] allowed us to determine that the (missing) articular sat between the caudal medial concavity of the surangular, the caudal dorsal concavity of the angular, and the caudal lateral concavity of the prearticular (Figure 1E).

The prearticular is a transversely compressed, sagittally elongate element, characterised by a sinuous profile in dorsal view. It is tallest rostrally and tapers caudally to a point, but maintains a fairly uniform transverse thickness along its length. The rostral two-thirds of the ventral margin are bevelled slightly laterally, presumably to abut against the caudal process of the splenial (presuming that the morphology of this element and the nature of its articulation were similar to that of *Giraffatitan* [25]). This ridge fades out on the caudal third of the ventral margin. The caudal portion of the prearticular is concave and faces dorsolaterally; this concavity would have been occupied by the (missing) articular in life. Further rostrally, the medial surface becomes slightly concave; the transition between the lateral and medial concavities occurs at the same point as the ventral ridge fades out.

#### Splenial (PMU 24705/1t [formerly PMU R 233 p]: Figure 20)

**Figure 20:**
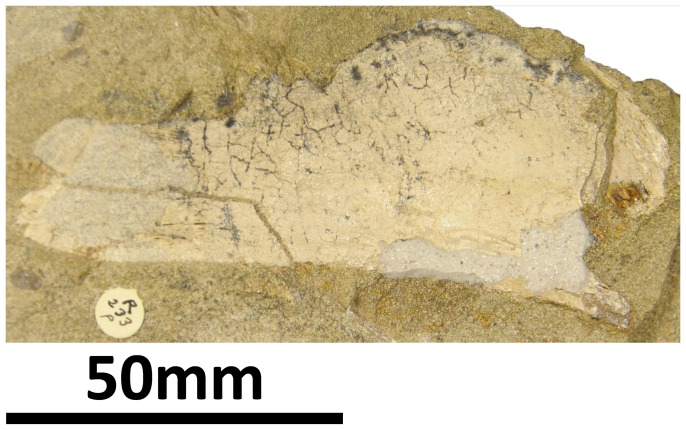
PMU 24705/1t (formerly PMU R 233 p), a probable splenial.

This flattened piece of bone has only been partially extracted from the rock in which it was preserved; unfortunately, it has been decided that it is too fragile to be removed, and the density difference between the bone and the matrix was not sufficient to separate them from each other in the CT data. Based on the size and morphology of this element, and knowing which elements are missing from the skull of *Euhelopus*, it is probable that this element is the right splenial, since it is a triangular, mediolaterally compressed and anteroposteriorly elongate element. This would suggest that the splenial of *Euhelopus* conformed to a morphology similar to that of *Giraffatitan* [25] and *Mamenchisaurus youngi* [9].

#### Teeth (PMU 24705/1 y [formerly “PMU M 2983”])

The teeth of *Euhelopus* were thoroughly described by Wilson and Upchurch [45], though the implications of a feature of these teeth which they identified was not fully realised.

The teeth of *Euhelopus* have small buttresses near the bases of the mesial and distal margins on the lingual side of each crown (as noted by Wilson and Upchurch [45]). However, the larger buttress is always the distal one. This means that loose left premaxillary-maxillary and right dentary teeth will have the buttress positioned in the same place, and the same will be true for right premaxillary-maxillary and left dentary teeth. Therefore, it is possible to narrow the position of any given loose *Euhelopus* tooth to two of four possible positions; in the case of PMU 24705, since both premaxillae-maxillae have (virtually) all alveoli occupied, the loose teeth can be confidently assigned to their respective dentaries. There are fifteen loose teeth in the PMU collection, all of which were mistakenly catalogued as PMU M 2983 (M stands for mammal, and they are clearly not mammalian - they are identical to the teeth set in the dentigerous elements of *Euhelopus*) until now – they are herein designated collectively as PMU 24705/1 y. Eight of these are interpreted to have come from the right dentary on the basis of the position of the lingual buttress, with the other seven representing left dentary teeth.

### Skull Reconstruction (Figure S1)

**Figure 21:**
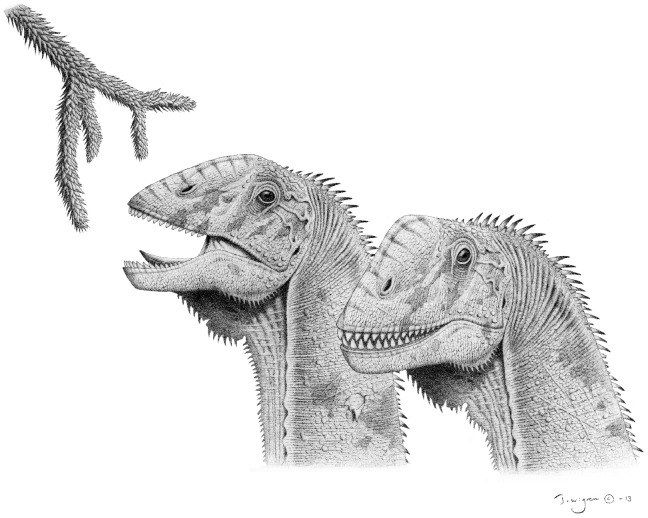
Reconstruction of the head of *Euhelopus zdanskyi* based on the three-dimensional reconstruction of the skull provided herein. Illustration by Tomas Wigren.

The skull of *Euhelopus* (Figure 1) closely resembles that of *Camarasaurus* [21], bearing some similarities to the skulls of *Mamenchisaurus youngi* [8], [9] and *Omeisaurus maioanus* [12] as well; this similarity was noted previously in a relative warps analysis [79]. The skulls of several non-sauropod sauropodomorphs (including *Yimenosaurus* Bai et al. 1990 [80] and *Aardonyx* Yates et al. 2010 [32]) and basal eusauropods (including *Shunosaurus* [7], *Turiasaurus* Royo-Torres et al. 2006 [81], [82] and *Jobaria* Sereno et al. 1999 [50]) are, overall, quite similar to those of the more derived *Euhelopus* and *Camarasaurus*. This skull shape, typified by being box-like, having a stepped “muzzle”, having large nares located rostrodorsal to the orbits, and having jaws lined with robust, slowly-replaced teeth, contrasts markedly with the skulls of titanosaurs and diplodocoids, which are elongate, lack a “muzzle”, have smaller nares retracted closer to the orbits, and have pencil-like, rapidly replaced teeth restricted to the front of the mouth [27], [73], [75], [83]. The contrast between the skull and dentition of *Euhelopus* (a basal somphospondyl) and titanosaurs (derived somphospondyls) suggests that titanosaurs filled a somewhat different niche to broad-toothed euhelopodids like *Euhelopus* (despite their close relationship), meaning that titanosaurs and the more narrow-toothed of the euhelopodids possibly competed with diplodocoids during the Cretaceous [84]. The skull and tooth morphology of *Euhelopus* strongly suggests that this animal fed upon hardy vegetation, and this interpretation, coupled with the postcranial morphology of the animal (specifically the long, flexible neck and subequal lengths of the humerus and femur [45], [85]), strongly supports a high-browsing lifestyle for *Euhelopus* (Figure 21).

## Conclusion

The rarity of sauropod skulls in the fossil record imbues each one known to science with great significance for phylogenetic and palaeoecological analyses. The skull elements of *Euhelopus* have been reassessed and illustrated to facilitate comprehensive comparisons with other sauropod cranial material. We have identified additional elements previously thought to be absent and revised the interpretations of some elements. Our final reconstruction of the skull, the most accurate for a non-titanosaurian somphospondylan produced to date, allows the articulations of the cranial elements to be viewed from virtually every angle.

## Supporting Information

Figure S1 **Three-dimensional reconstruction of the skull of** ***Euhelopus zdanskyi*** **.** Representative elements used in this reconstruction (digitally reflected to ensure symmetry): left premaxilla; left maxilla; right nasal; left lacrimal; right jugal; right postorbital; left squamosal; right quadratojugal; right quadrate; right pterygoid; right palatine; left dentary; left surangular; right angular; left prearticular.
(PDF)

Table S1 **List of sauropod species, their holotype and referred specimens and details of the cranial elements present in each specimen.** This list should not be viewed as comprehensive, and in some cases the reader is directed to sources which provide more detailed information of the cranial elements known for a particular species. This list does not include sauropod skull material which has not been assigned to a genus or species.
(XLSX)

Table S2 **List of the elements which comprise the holotype specimen of** ***Euhelopus zdanskyi*** **, PMU 24705 (formerly PMU R 233), held in the Palaeontological Museum, Museum of Evolution, Uppsala, Sweden.** An asterisk (*) indicates that a bone is figured as part of a reconstruction. Elements described by Young (1935) are currently lost and not included in this list.
(PDF)
