# Supplementary figures and images for: Photographic Atlas and Three-Dimensional Reconstruction of the Holotype Skull of Euhelopus zdanskyi with Description of Additional Cranial Elements
Source: PLoS One. 2013 Nov 21;8(11):e79932. doi: 10.1371/journal.pone.0079932 (PMC3836988; doi:10.1371/journal.pone.0079932)

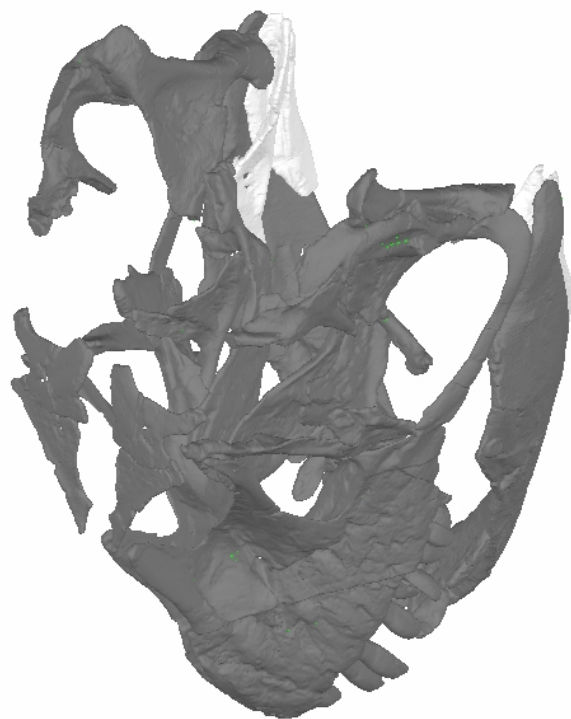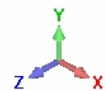

Click on the image to activate the 3D Model.

Supplement: Figure S1 — Three-dimensional reconstruction of the skull of Euhelopus zdanskyi . Representative elements used in this reconstruction (digitally reflected to ensure symmetry): left premaxilla; left maxilla; right nasal; left lacrimal; right jugal; right postorbital; left squamosal; right quadratojugal; right quadrate; right pterygoid; right palatine; left dentary; left surangular; right angular; left prearticular. (PDF) [file pone.0079932.s001.pdf]
