# Supplementary material for: Photographic Atlas and Three-Dimensional Reconstruction of the Holotype Skull of Euhelopus zdanskyi with Description of Additional Cranial Elements
Source: PLoS One. 2013 Nov 21;8(11):e79932. doi: 10.1371/journal.pone.0079932 (PMC3836988; doi:10.1371/journal.pone.0079932)
Supplement: Table S2 — List of the elements which comprise the holotype specimen of Euhelopus zdanskyi , PMU 24705 (formerly PMU R 233), held in the Palaeontological Museum, Museum of Evolution, Uppsala, Sweden. An asterisk (*) indicates that a bone is figured as part of a reconstruction. Elements described by Young (1935) are currently lost and not included in this list. (PDF) [file pone.0079932.s003.pdf]

Euhelopus zdanskyi (Wiman, 1929) Romer, 1956: holotype (PMU 24705; formerly PMU R 233) elements held in the Museum of Evolution, Uppsala, Sweden

Euhelopus zdanskyi holotype ("exemplar a")	PMU R 233	PMU 24705	Wiman, 1929		Mateer & McIntosh, 1985		Wilson & Upchurch, 2009	
**Element**	**Old number**	**New number**	**Description**	**Figure(s)**	**Description**	**Figure(s)**	**Description**	**Figure(s)**
**Premaxilla and maxilla (Left)**	PMU R 233 a	PMU 24705/1a	Pages 7-8: interpreted four teeth in premaxilla, one at premaxilla-maxilla suture, nine in maxilla.	Plate 1, fig. 2 (lateral*), 3 (anterior*) and 4 (dorsal*); Plate 2, fig. 1 (lateral) and 2 (medial)	-	Figure 5, a (lateral) and b (medial); Figure 6 (lateral*)	Page 207 (premaxilla): fully described - step-like offset of snout and lateral plate supporting teeth noted; Pages 207-208 (maxilla): fully described, preantorbital fenestra, lack of antorbital fossa and jugal articulation noted.	Figure 6 (reversed; medial)
**Premaxilla and maxilla (Right)**	PMU R 233 a	PMU 24705/1b	Pages 7-8: interpreted four teeth in premaxilla, one at premaxilla-maxilla suture, nine in maxilla.	Plate 1, fig. 1 (lateral*), 3 (anterior*) and 4 (dorsal*); Plate 2, fig. 3 (medial) and 4 (lateral)	-	Figure 5, c (medial) and d (lateral)	Page 207 (premaxilla): fully described - step-like offset of snout and lateral plate supporting teeth noted; Pages 207-208: fully described, preantorbital fenestra, lack of antorbital fossa and jugal articulation noted.	-
**Postorbital (Right)**	PMU R 233 a	PMU 24705/1g	Page 9: interpreted to be attached to part of postfrontal.	Plate 1, fig. 1 (lateral), 4 (dorsal), 9 (medial) and 10 (lateral)	-	Figure 6 (reversed; lateral*)	Page 208: fully described, incorrectly listed as the left element on page 207 but description only makes sense if it is the right.	-
**Squamosal (Left)**	PMU R 233 a	PMU 24705/1h	Page 9: interpreted as the right squamosal, depicted upside down.	Plate 1, fig. 1 (lateral; upside down*), 4 ("dorsal"; actually ventrolateral and upside down*); 7 (lateral; upside down) and 8 (medial; upside down)	Page 125-126: corrected Wiman's interpretation of this element – showed it was from the left side, not the right, and figured and mounted upside down by Wiman.	Figure 1, a (medial) and b (lateral): Figure 6 (lateral*)	Pages 208-209: fully described, noted as being incorrectly mounted (upside down as the right squamosal), interpreted the ventral process as broken	-
**Quadrate and pterygoid (Right)**	PMU R 233 a	PMU 24705/1k	Page 8-9: vomer not recognised; part of quadratojugal thought to be present.	Plate 1., fig. 1 (lateral*); Plate 2, fig. 5 (medial), 6 (lateral) and 7 (posterior)	Page 127: showed that the lateral flange of the quadrate was not the quadratojugal as had been interpreted by Wiman. Page 128: noted that the pterygoid was broken immediately posterior of the ectopterygoid process.	Figure 2, a (lateral; upside down) and b (medial; upside down); Figure 6 (reversed; lateral*)	Page 209 (quadrate): fully described, interpreted to have been vertically oriented and to have had a deep posterior fossa, but also that the lateral margin of the posterior fossa and the articular end are damaged. Page 209 (pterygoid): fully described, but description was limited by the preservation and reconstruction of this element	-

Euhelopus zdanskyi (Wiman, 1929) Romer, 1956: holotype (PMU 24705; formerly PMU R 233) elements held in the Museum of Evolution, Uppsala, Sweden

Euhelopus zdanskyi holotype ("exemplar a")	PMU R 233	PMU 24705	Wiman, 1929		Mateer & McIntosh, 1985		Wilson & Upchurch, 2009	
**Element**	**Old number**	**New number**	**Description**	**Figure(s)**	**Description**	**Figure(s)**	**Description**	**Figure(s)**
**Dentary and surangular (Left)**	PMU R 233 a	PMU 24705/1o	Page 10: Brief mention.	Plate 1, fig. 2 (lateral*), 3 (anterodorsal*) and 6 (medial)	Yes (surangular)	Figure 6 (lateral*)	Page 210 (dentary): fully described, U-shaped nature of the jaw highlighted; left surangular mentioned in specimen list on page 207	-
**Dentary and surangular (Right)**	PMU R 233 a	PMU 24705/1p	Page 10: Brief mention.	Plate 1, fig. 1 (lateral*), 3 (anterodorsal*), 5 (medial)	Yes (surangular, angular)	-	Page 210 (dentary): fully described; Page 210 (surangular): brief mention; right angular listed on page 207	-
**Axis**	PMU R 233 b	PMU 24705/2a	Page 12: Brief description.	Plate 3, fig. 1 (dorsal*), 2 (ventral*), 3 (left lateral*) and 5 (anterior)	-	-	Pages 211-212: fully described	Figure 7, a (left lateral) and b (right posterolateral)
**Cervical III**	PMU R 233 c	PMU 24705/3a	Pages 12-18: presacral vertebrae described in dedicated section	Plate 3, fig. 1 (dorsal*), 2 (ventral*), 3 (left lateral*), 6 (anterior) and 7 (posterior)	-	-	Pages 212-217 (postaxial cervical vertebrae): fully described	Figure 8 (right lateral)
**Cervical IV**	PMU R 233 d	PMU 24705/4	Pages 12-18: presacral vertebrae described in dedicated section	Plate 3, fig. 1 (dorsal*), 2 (ventral*), 3 (left lateral*) and 8 (anterior)	-	-	Pages 212-217 (postaxial cervical vertebrae): fully described; Pages 217-218 (ribs): fully described	
**Cervical V**	PMU R 233 e	PMU 24705/5	Pages 12-18: presacral vertebrae described in dedicated section	Plate 3, fig. 1 (dorsal*), 2 (ventral*) and 3 (left lateral*)	-	-	Pages 212-217 (postaxial cervical vertebrae): fully described; Pages 217-218 (ribs): fully described	
**Cervical VI**	PMU R 233 f	PMU 24705/6	Pages 12-18: presacral vertebrae described in dedicated section	Plate 3, fig. 1 (dorsal*), 2 (ventral*) and 3 (left lateral*)	-	-	Pages 212-217 (postaxial cervical vertebrae): fully described; Pages 217-218 (ribs): fully described	
**Cervical VII-VIII**	PMU R 233 g	PMU 24705/7a, 8	Pages 12-18: presacral vertebrae described in dedicated section	Plate 3, fig. 1 (dorsal*), 2 (ventral*), 3 (left lateral*) and 9 (VIII, posterior)	-	-	Pages 212-217 (postaxial cervical vertebrae): fully described; Pages 217-218 (ribs): fully described	Figure 9 (VIII, right lateral)
**Cervical rib VII (Left)**	PMU R 233 h	PMU 24705/7b	Pages 12-18: presacral vertebrae described in dedicated section	Plate 3, fig. 1 (dorsal*), 2 (ventral*) and 3 (left lateral*)	-	-	Pages 217-218 (cervical ribs): fully described	
**Cervicals IX-X**	PMU R 233 i	PMU 24705/9a, 10	Pages 12-18: presacral vertebrae described in dedicated section	Plate 3, fig. 1 (dorsal*), 2 (ventral*), 3 (left lateral*), 10 (IX, anterior) and 11 (X, posterior)	-	-	Pages 212-217 (postaxial cervical vertebrae): fully described; Pages 217-218 (ribs): fully described	Figure 10 (X, right lateral)
**Cervical XI**	PMU R 233 j	PMU 24705/11	Pages 12-18: presacral vertebrae described in dedicated section	Plate 3, fig. 1 (dorsal*), 2 (ventral*), 3 (left lateral*), 12 (anterior) and 13 (posterior)	-	-	Pages 212-217 (postaxial cervical vertebrae): fully described; Pages 217-218 (cervical ribs): fully described	

Euhelopus zdanskyi (Wiman, 1929) Romer, 1956: holotype (PMU 24705; formerly PMU R 233) elements held in the Museum of Evolution, Uppsala, Sweden

Euhelopus zdanskyi holotype ("exemplar a")	PMU R 233	PMU 24705	Wiman, 1929		Mateer & McIntosh, 1985		Wilson & Upchurch, 2009	
**Element**	**Old number**	**New number**	**Description**	**Figure(s)**	**Description**	**Figure(s)**	**Description**	**Figure(s)**
**Cervicals XII-XIV**	PMU R 233 k	PMU 24705/12-14	Pages 12-18: presacral vertebrae described in dedicated section	Plate 3, fig. 1 (dorsal*), 2 (ventral*), 3 (left lateral*), 14 (XII, anterior), 15 (XIV, posterior) and 16 (XIV, anterior)	-	-	Pages 212-217 (all postaxial cervical vertebrae): fully described; Pages 217-218 (ribs): fully described	Figure 11 (XIV, right lateral)
**Cervicals XV-XVII and dorsal I**	PMU R 233 l	PMU 24705/15-18	Pages 12-18: presacral vertebrae described in dedicated section	Plate 3, fig. 1 (dorsal*), 2 (ventral*), 3 (left lateral*) and 17 (dorsal I, posterior)	-	-	Pages 212-217 (postaxial cervical vertebrae): fully described; Pages 217-218 (ribs): fully described; Pages 218-219 (anterior dorsal vertebrae): fully described	Figure 11 (XV, right lateral); Figure 12 (XVI-XVII, right lateral)
**Dorsals II-VIII**	PMU R 233 m	PMU 24705/19-25	Pages 12-18: presacral vertebrae described in dedicated section	Plate 3, fig. 1 (dorsal*), 2 (ventral*), 3 (left lateral*), 4 (right lateral*) and 18 (II, anterior)	-	-	Pages 218-221: fully described	Figure 14 (VII, right lateral); Figure 15 (II-IV, dorsal); Figure 16 (II-V, right lateral); Figure 17 (VI, right lateral); Figure 18 (VII, right lateral)
**Femur (Left)**	PMU R 233 n	PMU 24705/28	Page 18: described as having a short well-developed fourth trochanter and spongy internal bone texture	Plate 3, fig. 20 (posterior), 21 (lateral), 22 (anterior) and 23 (proximal)	-	-	Pages 223-224: fully described	
**Dorsal rib III (Left)**	PMU R 233 o	PMU 24705/27a	Page 18: brief mention; pages 22-23: brief description	Plate 3, fig. 18 (anterior) and 19 (posterior)	-	-	Page 221: fully described	Figure 21 (posterior; proximal end only)
**?Splenial (?Right)**	PMU R 233 p	PMU 24705/1t	-	-	-	-	-	-
**Rock associated with skull block**	PMU R 233 q	PMU 24705/1x	-	-	-	-	-	-
**Prearticular (Left)**	PMU R 233 r	PMU 24705/1r	-	-	Page 128: described as a left and as being similar to the prearticular of *Camarasaurus*	Figure 4, a (lateral) and b (medial)	Page 207: mentioned in specimen list	-
**Angular (Right)**	PMU R 233 s	PMU 24705/1q	-	-	Page 128: described as the right angular, said to closely resemble the angular of *Camarasaurus*	Figure 4, c ("upper" = medial) and d ("lower" = lateral)	Page 210: described as the left angular, absence of evidence for mandibular fenestra noted	-
**Nasal and Prefrontal (Right; caudal portion)**	PMU R 233 t	PMU 24705/1d	-	-	Page 125: described as the right frontal	Figure 1, c (dorsal) and d (ventral)	Page 208: fully described as left nasal, interpreted as having anteromedial and anterolateral processes	-
**Jugal (Right)**	PMU R 233 u	PMU 24705/1u	-	-	-	-	-	-
**Nasal (Right; rostral portion)**	PMU R 233 v	PMU 24705/1c	-	-	-	-	-	-

Euhelopus zdanskyi (Wiman, 1929) Romer, 1956: holotype (PMU 24705; formerly PMU R 233) elements held in the Museum of Evolution, Uppsala, Sweden

***Euhelopus zdanskyi* holotype ("exemplar a")**	**PMU R 233**	**PMU 24705**	**Wiman, 1929**		**Mateer & McIntosh, 1985**		**Wilson & Upchurch, 2009**	
**Element**	**Old number**	**New number**	**Description**	**Figure(s)**	**Description**	**Figure(s)**	**Description**	**Figure(s)**
	PMU R 233 w						-	-
**Quadratojugal (Right; dorsal process)**	PMU R 233 x	PMU 24705/1s	-	-	-	-	-	-
**Postorbital (Right; jugal process)**	PMU R 233 y	PMU 24705/1v	-	-	-	-	-	-
**Quadratojugal (Left)**	PMU R 233 z	PMU 24705/1i	-	-	Page 127: identified and likened to the quadratojugal of *Camarasaurus* and *Giraffatitan* (*Brachiosaurus*).	Figure 3, e (medial)	Page 209: fully described, both quadratojugals interpreted as having the tops of their anterodorsal processes (which may have contacted the squamosal) missing	-
**Quadratojugal (Right)**	PMU R 233 å	PMU 24705/1j	-	-	Page 127: identified and likened to the quadratojugal of *Camarasaurus* and *Giraffatitan* (*Brachiosaurus*).	Figure 3, f (medial); Figure 6 (reversed; lateral*)	Page 209: fully described, both quadratojugals interpreted as having the tops of their anterodorsal processes (which may have contacted the squamosal) missing	-
**Pterygoid (Left; partial)**	PMU R 233 ä	PMU 24705/1l	-	-	-	-	-	-
**Pterygoid (Left; partial)**	PMU R 233 ö	PMU 24705/1w	-	-	-	-		-
**Lacrimal (Left)**	PMU R 233 α	PMU 24705/1e	-	-	Page 127: identified and briefly described; right is incorrectly stated to be the more complete	Figure 3, c (medial) and d (lateral); Figure 6 (upside down; lateral*)	Page 207: mentioned in specimen list	
**Lacrimal (Right)**	PMU R 233 β	PMU 24705/1f	-	-	Page 127: identified and briefly described; right is incorrectly stated to be the more complete	Figure 3, a (anterolateral; upside down) and b (posterolateral, upside down)	Page 207: mentioned in specimen list	-
**Palatine (Right)**	PMU R 233 γ	PMU 24705/1n	Page 8: described as a vomer.	Plate 2, fig. 8 (medial), 9 (ventral) and 10 (lateral).	Page 127: identified as vomer, briefly described.	Figure 2, c (lateral) and d (medial)	Page 209: fully described.	-
**Palatine (Left)**	PMU R 233 δ	PMU 24705/1m	Page 8: described as a vomer.	Plate 2, fig. 11 (medial)	Page 127: identified as vomer, briefly described.	-	Page 209: fully described.	-
**Dorsal rib V (Left; proximal)**	PMU R 233 ε	PMU 24705/27b	-	-	-	-	Page 221: incorrectly stated as absent	-
**Dorsal rib (Left; proximal)**	PMU R 233 ζ	PMU 24705/27c	-	-	-	-	Page 221: incorrectly stated as absent	-
**Cervical rib II (proximal)**	PMU R 233 η	PMU 24705/2b	-	-	-	-	Page 217: incorrectly stated as absent	-
**Cervical rib III (Right)**	PMU R 233 ϑ	PMU 24705/3c	-	-	-	-	Page 217: incorrectly stated not to be present	-
**Cervical ribs (distal) in matrix**	PMU R 233 ι	PMU 24705/26h	-	-	-	-	-	-
**Cervical rib (distal)**	PMU R 233 κ	PMU 24705/26a	-	-	-	-	Pages 217-218 (cervical ribs): fully described	-
**Cervical rib (distal)**	PMU R 233 λ	PMU 24705/26b	-	-	-	-	Pages 217-218 (cervical ribs): fully described	-

Euhelopus zdanskyi (Wiman, 1929) Romer, 1956: holotype (PMU 24705; formerly PMU R 233) elements held in the Museum of Evolution, Uppsala, Sweden

Euhelopus zdanskyi holotype ("exemplar a")	PMU R 233	PMU 24705	Wiman, 1929		Mateer & McIntosh, 1985		Wilson & Upchurch, 2009	
**Element**	**Old number**	**New number**	**Description**	**Figure(s)**	**Description**	**Figure(s)**	**Description**	**Figure(s)**
**Cervical rib (distal)**	PMU R 233 μ	PMU 24705/26c	-	-	-	-	Pages 217-218 (cervical ribs): fully described	-
**Cervical rib (distal)**	PMU R 233 ν	PMU 24705/26d	-	-	-	-	Pages 217-218 (cervical ribs): fully described	-
**Cervical rib (distal)**	PMU R 233 ξ	PMU 24705/26e	-	-	-	-	Pages 217-218 (cervical ribs): fully described	-
**Cervical rib (distal)**	PMU R 233 o	PMU 24705/26f	-	-	-	-	Pages 217-218 (cervical ribs): fully described	-
**Cervical rib III (Left)**	PMU R 233 ϝ	PMU 24705/3b	-	-	-	-	Page 217: incorrectly stated as absent	-
**Cervical rib IX (Left; distal)**	-	PMU 24705/9b	-	-	-	-	Pages 217-218 (cervical ribs): fully described	-
**Cervical rib (distal)**	-	PMU 24705/26g	-	-	-	-	Pages 217-218 (cervical ribs): fully described	-
**Cervical rib in matrix**	-	PMU 24705/26i	-	-	-	-	-	-
**Dorsal rib VII (mid-shaft)**	-	PMU 24705/27d	-	-	-	-	Page 221: incorrectly stated as absent	-
**Unidentified element (fragmentary)**	-	PMU 24705/29	-	-	-	-	-	-
**Unidentified large element (fragmentary)**	-	PMU 24705/30	-	-	-	-	-	-
**Eight loose teeth with buttress at bottom right when oriented with crown dorsally and root ventrally (i.e. left premaxillary or maxillary teeth, or right dentary teeth); likely to be mostly, if not exclusively, right dentary teeth since the left premaxilla and maxilla have few if any vacant alveoli**	"PMU M 2983"	PMU 24705/1y	Page 10: brief mention	Plate 2, fig. 18; 19-20; 21-22; 23.	-	-	Pages 210: fully described, dental formula = premaxilla 4; maxilla 10; dentary 13; occlusal pattern, wear, enamel texture and lingual buttresses all noted	-
**Seven teeth with buttress at bottom left when oriented with crown dorsally and root ventrally (i.e. right premaxillary or maxillary teeth, or left dentary teeth); likely to be mostly, if not exclusively, left dentary teeth since the right premaxilla and maxilla have few vacant alveoli**	"PMU M 2983"	PMU 24705/1y	Page 10: brief mention	Plate 2, fig. 12-13, 24; 14; 15; 16; 17.	-	-	Pages 210: fully described, dental formula = premaxilla 4; maxilla 10; dentary 13; occlusal pattern, wear, enamel texture and lingual buttresses all noted	-

An asterisk (*) indicates that a bone is figured as part of a reconstruction. Elements described by Young (1935) are currently lost and not included in this list.

References

Mateer, N.J., McIntosh, J.S., 1985. A new reconstruction of the skull of *Euhelopus zdanskyi* (Saurischia: Sauropoda). **Bulletin of the Geological Institution of the University of Uppsala (new series)** 11, 124-132.

Wilson, J.A., Upchurch, P., 2009. Redescription and reassessment of *Euhelopus zdanskyi* (Dinosauria: Sauropoda) from the Early Cretaceous of China. **Journal of Systematic Palaeontology** 7, 199-239.

Wiman, C., 1929. Die Kreide-Dinosaurier aus Shantung [The Cretaceous Dinosaur from Shantung]. **Palaeontologia Sinica (Series C)** 6, 1-67.
